# Endoscopic Modified Medial Maxillectomy for Resection of an Inverted Papilloma Originating from the Entire Circumference of the Maxillary Sinus

**DOI:** 10.1155/2015/952923

**Published:** 2015-06-03

**Authors:** Kota Wada, Takashi Ishigaki, Yutaro Ida, Yuki Yamada, Sachiko Hosono, Hideo Edamatsu

**Affiliations:** Department of Otorhinolaryngology, Toho University, 6-11-1 Omori-Nishi, Ota-ku, Tokyo 143-8541, Japan

## Abstract

For treatment of a sinonasal inverted papilloma (IP), it is essential to have a definite diagnosis, to identify its origin by computed tomography (CT) and magnetic resonance imaging (MRI), and to select the appropriate surgical approach based on the staging system proposed by Krouse. Recently, a new surgical approach named endoscopic modified medial maxillectomy (EMMM) was proposed. This approach can preserve the inferior turbinate and nasolacrimal duct. We successfully treated sinonasal IP with EMMM in a 71-year-old female patient. In this patient, the sinonasal IP originated from the entire circumference of the maxillary sinus. EMMM is not a difficult procedure and provides good visibility of the operative field. Lacrimation and empty nose syndrome do not occur postoperatively as the nasolacrimal duct and inferior turbinate are preserved. EMMM is considered to be a very favorable approach for treatment of sinonasal IP.

## 1. Introduction

Sinonasal inverted papillomas (IPs) are one of most commonly found benign tumors in the paranasal sinuses [1]. Inverted papilloma may become malignant or complicated with cancer [2, 3] and complete surgical resection is therefore essential for treatment. Before surgery, IP must be diagnosed based on distinctive clinical and imaging findings of the nasal cavity. Once the condition has unquestionably been diagnosed as IP, the appropriate approach can be selected. Many reports state that the range of the lesion must be defined, and the surgical approach must be selected based on the staging system proposed by Krouse [4, 5]. It is also understood that the origin of the tumor is very important.

A computed tomography (CT) scan of the paranasal sinus may reveal localized thickening of the bone [6, 7], indicating the site of origin of IP. In addition, a magnetic resonance imaging (MRI) scan may show as the origin of IP, an inflammatory change on the opposite site of the unoccupied area, or a site where a serpentine cerebriform filamentous structure converges [8]. The surgical procedure is selected based on the Krouse staging system and the site of origin of the tumor. Commonly used procedures include endoscopic sinus surgery (ESS), Caldwell-Luc surgery, endoscopic medial maxillectomy (EMM), and lateral rhinotomy (LR). If IP originated from the frontal sinus, an endoscopic modified Lothrop procedure (EMLP) may be selected.

EMMM was recently proposed by Nakayama et al. as a surgical treatment for sinonasal IP [9]. In this approach, the maxillary sinus is operated upon, while the inferior turbinate and nasolacrimal duct are preserved. In this report, we describe our experience of successfully utilizing EMMM to resect IP in the maxillary sinus that originated from the entire circumference of the maxillary sinus.

## 2. Case Presentation

**Figure 1:**
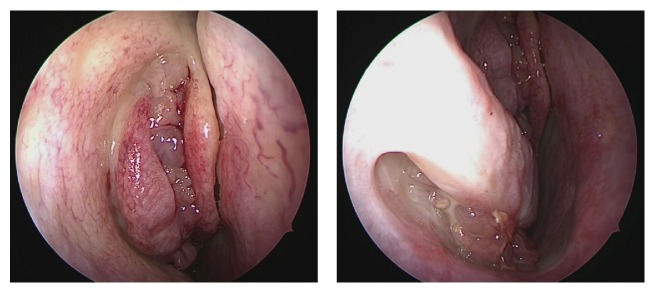
Preoperative observation of nasal cavity. Inverted papilloma is observed behind the uncinate process and inferior meatus.

**Figure 2:**
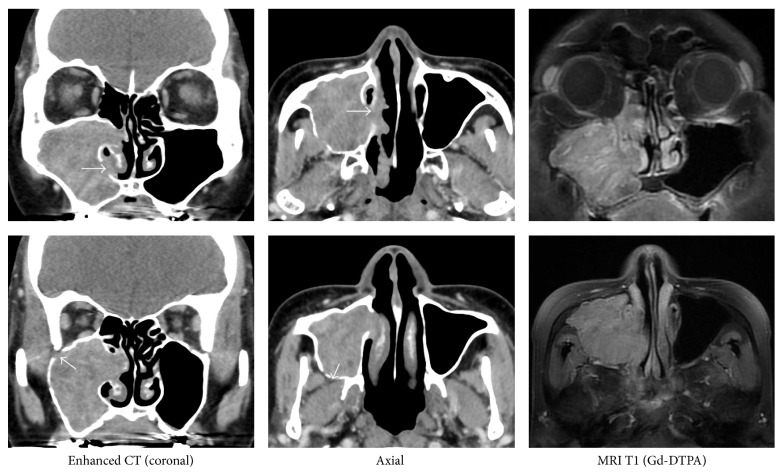
CT and MRI Imaging. The contrast CT shows bone defects in the anterior and medial walls of the maxillary sinus. There are no indications that the thickening of the bone should be suspected as the site of origin. MRI T1 (Gd-DTPA) shows secondary maxillary sinusitis and a serpentine cerebriform filamentous structure, but there is no mass to otherwise indicate a possible origin.

**Figure 3:**
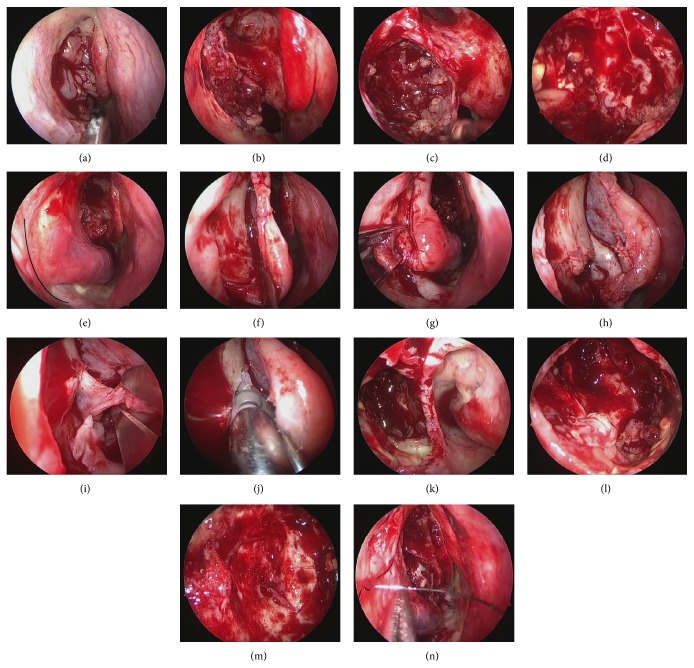
Endoscopic Modified Medal Maxillectomy (EMMM). (a) The uncinate process was resected, and an IP deviating from the maxillary sinus was observed. (b) The uncinate process and ethmoidal bulla were resected, and the maxillary sinus was opened. A tumor was found to be filling the maxillary sinus. (c) Inverted papilloma in the maxillary sinus was debulked. All of the IPs were adhering to the posterior wall. (d) The anterior and inferior walls of the maxillary sinus were not visible when viewed from the fontanelle of the maxillary sinus with a 70-degree endoscope. (e) From the anterior portion of the inferior turbinate behind the pyriform aperture, the mucosa was incised towards the floor of nasal cavity (black line). (f) The mucosa of inferior turbinate was detached. The mucosa on the inferior meatus was detached to expose the conchal crest. (g) The conchal crest was cut with a chisel, the inferior turbinate bone was disengaged, and the inferior turbinate was moved medially. (h) When the inferior turbinate was moved medially, the conchal crest and the inferior end of the nasolacrimal duct (∗) could be observed. (i) The nasolacrimal duct was identified, and the IP that was deviating to the inferior meatus was resected. (j) The lacrimal process of the inferior turbinate, frontal process of the maxilla, and inferior portion of the lacrimal bone were drilled with 2.5 mm Curved Diamond DCR Bur (Medtronic), in order to make the superior portion of the lacrimal duct visible. (k) The inside of the maxillary sinus was visible from the inferior meatus side. (l) Inverted papilloma was observed on the anterior and inferior walls. (m) All of the IPs found on the anterior and inferior walls were resected with the 70-degree endoscope. (n) The pyriform aperture and mucosa of the inferior turbinate were sutured and the surgery was completed.

**Figure 4:**
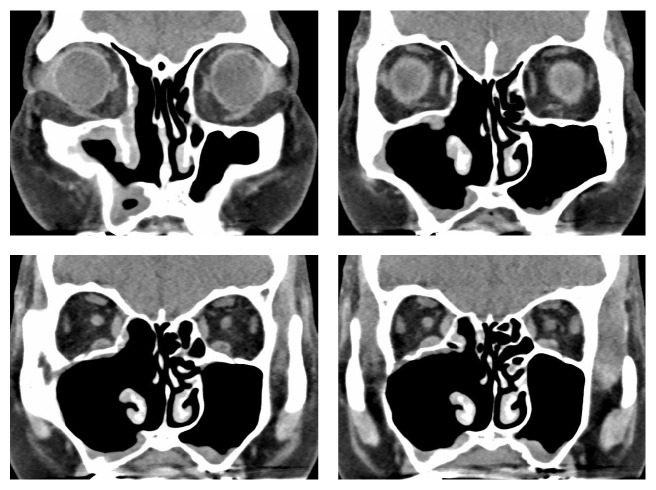
Postoperative CT observation. Granulation-like mucosal edema remained, but the maxillary sinus was widely opened from the middle and inferior meatus sides. The inferior turbinate was preserved.

**Figure 5:**
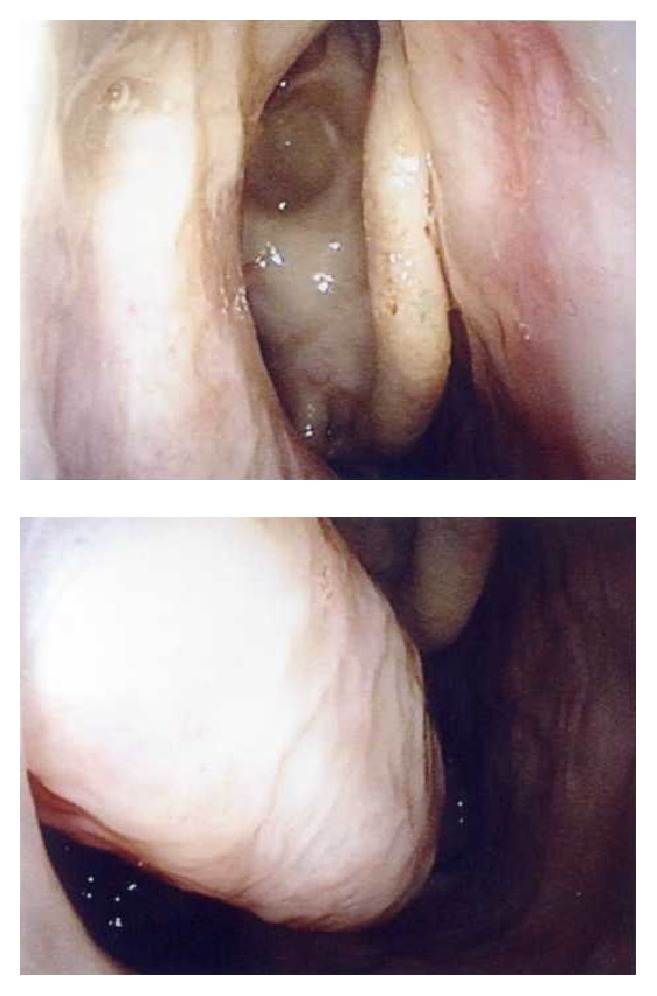
Postoperative observation of nasal cavity. The inferior turbinate was found in its inherent position.

A 71-year-old woman visited an otorhinolaryngologist with 5-year history of nasal congestion. She was diagnosed as having a nasal polyp. Since IP was found at biopsy, she was referred to our hospital in order to undergo surgery. In the nasal cavity, the tumor that was suspected to be IP was observed in the middle and inferior meatus (Figure 1). A CT scan of the paranasal sinus revealed a shadow that occupied the maxillary sinus and deviated to the ethmoidal sinus and a defect in the posterior wall and medial bone of the maxillary sinus (Figure 2). Preoperative squamous cell carcinoma (SCC) antigen level was high at 11.0 ng/mL. We suspected a cancerous change of IP or a complication of cancer and performed another biopsy, concluding that the condition was IP. The lesion was examined by CT and MRI to locate the origin of IP. The CT did not reveal localized thickening of the bone, and the MRI did not show secondary changes in the maxillary sinus or a distinctive mass of serpentine cerebriform filamentous structure (Figure 2). Findings revealed erosion or defects of the bone in the posterior and medial walls of the maxillary sinus, but the origin of IP could not be identified. It was believed that IP originated from a wide area. The patient refused to undergo lateral rhinotomy and was therefore informed that ESS and EMMM procedures would be used concomitantly and that a transantral approach (TA), Coldwell-Luc surgery, would be used if necessary. The patient gave her consent. The surgery was performed under general anesthesia. The uncinate process was removed and IP was found to be deviating into the nasal cavity and the normal mucosa in the anterior ethmoidal sinus. The frontal and maxillary sinuses were opened wide, while the posterior ethmoidal sinus was not opened. When observed at a 70-degree endoscope, the tumor in the maxillary sinus was seen to have adhered to the posterior, superior, and medial walls and the origin of IP was widely extensive, as anticipated preoperatively. The anterior and inferior lesions could not have been sufficiently resected if approached from the fontanelle of the maxillary sinus. Therefore, EMMM was selected (Figure 3). Slightly posterior to the pyriform aperture, the mucosa was incised from the superior portion of the inferior turbinate towards the nasal floor, and the nasal mucosa was elevated from the lateral wall of the nasal cavity. The mucosa on the lateral side of the inferior meatus was detached from the medial side of the maxillary sinus. The inferior turbinate bone was observed endoscopically and the conchal crest was cut with a chisel, allowing for the inferior portion of the nasolacrimal duct to be observed and for the inferior turbinate bone to be freely moved medially. As the nasolacrimal duct could be clearly observed by endoscope, the tumor deviating to the inferior meatus and the lateral mucosa and the bony wall of the inferior meatus could be sufficiently resected. The lacrimal process of the inferior turbinate, the frontal process of the maxilla, and the inferior portion of the lacrimal bone were ground with a 2.5-mm Curved Diamond DCR Bur (Medtronic), which made superior portion of lacrimal duct visible. Maxillary sinus was widely opened from the inferior meatus side so that endoscope could be inserted from inferior meatus towards maxillary sinus. The anterior, inferior, and medial walls of the maxillary sinus could be observed endoscopically. Inverted papilloma had adhered to the anterior, medial, and inferior walls; that is, it adhered to the entire circumference of the maxillary sinus. It was considered that there were all IP-involved mucosae without normal mucosa inside the maxillary sinus. The posterior portion could be observed with a 0-degree endoscope and the anterior portion could be observed by 70-degree endoscope. Inverted papilloma could be resected with highly curved forceps. The pyriform aperture and the mucosa of the inferior turbinate were sutured and the surgery was completed. Since part of the tumor had eroded the bone of the posterior wall, destroyed the inferior meatus, and deviated to the nasal cavity, a complication of cancer was suspected and an intraoperative consultation was performed. No malignant finding was obtained. In this case, there was no thickening of the bony wall of posterior wall of the maxillary sinus. We did remove the mucosa only without thinning of the bony wall of the maxillary sinus. The patient was free from recurrence 3 months after the surgery (Figure 4). The levels of SCC antigen had decreased to 2.9 ng/mL. Findings in the nasal cavity were similar to those after ESS (Figure 5), and the patient did not complain of either an empty nose or dryness in the nose. The patient had mild numbness around the lips, but no symptom in the eyes, such as lacrimation.

## 3. Discussion

The importance of surgery in the treatment of IP is unquestionable. Recently, endoscopy has played an important role in surgeries of the paranasal sinuses. ESS is often selected as an appropriate procedure for surgical treating IP [10]. En block resection is desirable, but this is difficult with an endoscope owing to the limited visibility of the operative field. In endoscopic surgery, it is important to identify the origin of the tumor and the surgical margin, by piecemeal resection and debulking surgery [8]. Krouse suggested a staging system based on the range of IP and further suggested the procedures that should be selected for IP at different stages [4]. For IP in the maxillary sinus, ESS is recommended for stage T2, in which lesions exist on the medial side and the superior wall of the maxillary sinus. EMM or TA is recommended for stage T3, in which lesions exist on the lateral side and the inferior, posterior, and anterior walls of the maxillary sinus. Extended surgery, such as surgery for a malignant tumor, is recommended for T4 in which the tumor extends outside of maxillary sinus. However, we consider ESS to be a viable option for treating tumors that originate from the lateral side and the posterior wall of the maxillary sinus as ESS provides a good visibility of the operative field. However, ESS is not a good choice for tumors that originate from the anterior and inferior walls of the maxillary sinus. LR requires a skin incision. EMM is an endonasal surgical procedure but does not appear to be a desirable approach because it necessitates the resection of the inferior turbinate and the cutting of the nasolacrimal duct, which impairs the function of the nose and may induce postoperative lacrimation [11].

We selected EMMM because it allows for the preservation of the physiological function of the nose. Nakayama et al. reported that EMMM was effective for treating an odontogenic tumor or a cyst found in the inferior portion of the maxillary sinus, the mucocele of the maxillary sinus found on the lateral side of the nasolacrimal duct, or IP originating from the anterior wall of the maxillary sinus [12]. This method is obviously different from Ghosh's method, because their methods have to perform inferolateral, lateral, and superolateral osteotomy from the pyriform aperture and remove the inferior turbinate [13]. In this case, we used EMMM (Nakayama's method) for IP that originated from the entire circumference of the maxillary sinus. The inferior turbinate and the nasolacrimal duct could be moved to the medial side. A good visibility could be achieved and the tumor deviating to the inferior meatus could be sufficiently resected without any problems. The lateral side, the anterior wall, and the tumor on the anterior wall could be observed, and the tumor could be resected under a 70-degree endoscope. The tumor on the inferior wall could be observed as the lateral wall of the inferior meatus was resected down to the floor of the nasal cavity. In EMMM, a 0-degree endoscopy can provide adequate visibility of the lesion, and a 70-degree side-viewing endoscopy allows the entire circumference of the maxillary sinus to become visible. The lateral anterior side of the nasolacrimal duct (recessus prelacrimalis) was especially visible, being rarely observed in the Caldwell-Luc approach.

In order to prevent recurrence, IP must be totally resected using a microdebrider, suction, or cutting forceps. Visibility and resectability are different. An extended tumor with a small origin is not difficult to resect. When a tumor originates from a wide area, like that observed in our patient, it is important to use EMMM to allow the inside of the maxillary sinus to become visible, to control bleeding, to select forceps that can fully resect the lesion, and to ensure that the tumor has been fully resected.

It has been reported that the incidence of recurrence of sinonasal IP following surgery is about 12% [14]. Endoscopic surgery without sufficient visibility and an extended tumor origin are considered to be risk factors of recurrence. In our patient, the origin of the tumor extended to the entire circumference of the maxillary sinus, and bone defects were present in some parts of the medial and posterior walls of the maxillary sinus. It is reported that 5 to 15% of IPs progress to malignant disease or are complicated with cancer [15]. Our patient presented with a high preoperative SCC antigen level of 11.0 ng/mL. Some reports have indicated that the SCC antigen is useful for the diagnosis of IP [16]. Our patient had a wide tumor origin, bone defects, and high preoperative levels of SCC antigen. Careful follow-up is essential to prevent recurrence or malignancy following surgery.

## 4. Conclusion

We used EMMM to surgically treat an IP that originated from the entire circumference of the maxillary sinus. All walls of the maxillary sinus could be observed from the inferior meatus side, and the tumor was sufficiently resected. The inferior turbinate and the nasolacrimal duct could be preserved because they could be moved medially, which prevented lacrimation or empty nose syndrome, with a good postoperative clinical course. Our experience indicates that EMMM is an effective and relatively easy approach for treating IP that originated in the anterior, inferior, and medial walls of the maxillary sinus.

## References

[1] Buchwald C., Nielsen L. H., Nielsen P. L., Ahlgren P., Tos M. (1989). Inverted papilloma: a follow-up study including primarily unacknowledged cases. *American Journal of Otolaryngology*.

[2] Lesperance M. M., Esclamado R. M. (1995). Squamous cell carcinoma arising in inverted papilloma. *Laryngoscope*.

[3] Mirza S., Bradley P. J., Acharya A., Stacey M., Jones N. S. (2007). Sinonasal inverted papillomas: recurrence, and synchronous and metachronous malignancy. *Journal of Laryngology and Otology*.

[4] Krouse J. H. (2000). Development of a staging system for inverted papilloma. *The Laryngoscope*.

[5] Oikawa K., Furuta Y., Nakamaru Y., Oridate N., Fukuda S. (2007). Preoperative staging and surgical approaches for sinonasal inverted papilloma. *Annals of Otology, Rhinology and Laryngology*.

[6] Chiu A. G., Jackman A. H., Antunes M. B., Feldman M. D., Palmer J. N. (2006). Radiographic and histologic analysis of the bone underlying inverted papillomas. *Laryngoscope*.

[7] Yousuf K., Wright E. D. (2007). Site of attachment of inverted papilloma predicted by CT findings of osteitis. *The American Journal of Rhinology*.

[8] Iimura J., Otori N., Ojiri H., Moriyama H. (2009). Preoperative magnetic resonance imaging for localization of the origin of maxillary sinus inverted papillomas. *Auris Nasus Larynx*.

[9] Nakayama T., Asaka D., Okushi T., Yoshikawa M., Moriyama H., Otori N. (2012). Endoscopic medial maxillectomy with preservation of inferior turbinate and nasolacrimal duct. *American Journal of Rhinology and Allergy*.

[10] Reh D. D., Lane A. P. (2009). The role of endoscopic sinus surgery in the management of sinonasal inverted papilloma. *Current Opinion in Otolaryngology and Head and Neck Surgery*.

[11] Nakamaru Y., Furuta Y., Takagi D., Oridate N., Fukuda S. (2010). Preservation of the nasolacrimal duct during endoscopic medial maxillectomy for sinonasal inverted papilloma. *Rhinology*.

[12] Nakayama T., Otor N., Asaka D., Okushi T., Haruna S. (2014). Endoscopic modified medial maxillectomy for odontogenic cysts and tumours. *Rhinology*.

[13] Ghosh A., Pal S., Srivastava A., Saha S. (2015). Modification of endoscopic medial maxillectomy: a novel approach for inverted papilloma of the maxillary sinus. *The Journal of Laryngology & Otology*.

[14] Busquets J. M., Hwang P. H. (2006). Endoscopic resection of sinonasal inverted papilloma: a meta-analysis. *Otolaryngology—Head and Neck Surgery*.

[15] Lesperance M. M., Esclamado R. M. (1995). Squamous cell carcinoma arising in inverted papilloma. *The Laryngoscope*.

[16] Suzuki M., Deng Z., Hasegawa M., Uehara T., Kiyuna A., Maeda H. (2012). Squamous cell carcinoma antigen production in nasal inverted papilloma. *The American Journal of Rhinology and Allergy*.

